# Moving in extreme environments: inert gas narcosis and underwater activities

**DOI:** 10.1186/s13728-014-0020-7

**Published:** 2015-02-24

**Authors:** James E Clark

**Affiliations:** Centre of Human & Aerospace Physiological Sciences and British Heart Foundation Excellence Centre, Cardiovascular Division, St Thomas’ Hospital, King’s College London, London, SE1 7EH UK

**Keywords:** Extreme environments, Narcosis, Inert gas, Nitrogen, SCUBA, Diving, Underwater

## Abstract

Exposure to the underwater environment for pleasure or work poses many challenges on the human body including thermal stress, barotraumas, decompression sickness as well as the acute effects of breathing gases under pressure. With the popularity of recreational self-contained underwater breathing apparatus (SCUBA) diving on the increase and deep inland dive sites becoming more accessible, it is important that we understand the effects of breathing pressurised gas at depth can have on the body. One of the common consequences of hyperbaric gas is the narcotic effect of inert gas. Nitrogen (a major component of air) under pressure can impede mental function and physical performance at depths of as little as 10 m underwater. With increased depth, symptoms can worsen to include confusion, disturbed coordination, lack of concentration, hallucinations and unconsciousness. Narcosis has been shown to contribute directly to up to 6% of deaths in divers and is likely to be indirectly associated with other diving incidents at depth. This article explores inert gas narcosis, the effect on divers’ movement and function underwater and the proposed physiological mechanisms. Also discussed are some of the factors that affect the susceptibility of divers to the condition. In conclusion, understanding the cause of this potentially debilitating problem is important to ensure that safe diving practices continue.

## Review

### Background

According to some reports, recreational diving using self-contained underwater breathing apparatus (SCUBA) is an increasingly popular sport throughout the world. It is estimated that there are over 7 million qualified SCUBA divers, with up to 500,000 new divers being certified every year worldwide [1,2]. How many of these newly qualified divers who continue to dive is hard to determine since no single authority is able to publish figures. In addition to those enjoying diving as a hobby, there is a body of professionals exposed to similar environments (oil and gas industry, rescue, scientific and archaeology, engineers and diving chamber workers), which the *Bureau of Labor Statistics (US)* estimates to be around 3,600 in America [3].

Diving includes a number of factors that can affect function and movement and endanger divers’ health. SCUBA diving is, however, a very safe sport and there are, on average, less than 20 deaths per 100,000 divers (0.02%) annually according to the Divers Alert Network (DAN), meaning diving has a similar risk to most other forms of regular exercise [4,5]. Individuals are exposed to water temperatures than can result in progressive heat loss [6], bulky thermal protection can impede physical activity and there is a risk of entrapment or entanglement due to the bulky equipment carried [7]. Part of the risk involved in diving is the increase in ambient pressure when the body enters the underwater environment. For every 10 metres of sea water (msw) depth, there is a net increase of 1 atmosphere (atm) of ambient pressure; such that at 10 msw, the body is exposed to 2 atm, and at 30 msw 4 atm. Using SCUBA equipment, the diver receives compressed gas (usually air) at ambient pressure through a mouthpiece. Therefore, as a diver descends, they are exposed to increased inspired gas pressures, the consequences of which are not trivial. Understanding the consequences of hyperbaric exposure requires the application and knowledge of complex physiological processes more than other environments in which humans move [8]. The hyperbaric environment carries the risks of barotrauma, decompression sickness and equipment failure resulting in suffocation or drowning; the results of which can be life-changing [9]. The physiological effect of hyperbaric gases on SCUBA divers can loosely be divided into those resulting from prolonged exposure such as decompression illness (DCI) and the immediate, acute, effects such as oxygen toxicity and the narcotic effects of inert gases which are the focus of this review [2,9-11].

One of the first reports of what is now known as inert gas narcosis (IGN) was by Colladon, a French physician who, in 1826, descended to 20 msw in a diving bell. He described “…*a state of excitement as if I had drunk some alcoholic liquor…*” [12]. Over the subsequent century, there were a number of reports of healthy divers becoming ‘mentally or emotionally abnormal’ when diving to depth (approximately 100 msw) and many of their symptoms were incorrectly attributed to impurities in the breathing mixture [2]. In 1935, Beknke and co-workers first suggested that nitrogen gas might have been the mediator of the observed behaviour, by utilising different gas breathing mixtures in their experiments [13]. Many have experienced the phenomenon of IGN but it is still poorly understood and managed.

### Current guidelines on exposure to hyperbaric gas

The international diving agencies (such as the Professional Association of Diving Instructors, PADI and the British Sub-Aqua Club, BSAC) try to mitigate the exposure to hyperbaric nitrogen by limiting the depths to which recreational divers can dive without additional training or equipment [14-16]. The Health and Safety Executive (HSE, UK) issues guidelines on the exposure limits for air diving operations; however, these consider only depths and durations for decompression requirements and the US Navy Diving Manual discusses narcosis in the context of adequate training [17,18]. With increasing depth, there is increased risk. With an understanding about the onset of significant IGN in scuba diving, it is not at all surprising that most international sport diving qualifications have a depth limit of around 30 msw [14,15].

### Narcosis and injury or death in divers

The Australian diving fatality database (Project Stickybeak) estimates that nitrogen narcosis contributed to approximately 9% of deaths reported and, in the UK, DAN cites 3.6% of reported deaths to have been caused by IGN in 2010 [2,7]. Depth alone (without direct evidence for narcosis) was shown to have contributed to 54.3% of advanced open-water training fatalities worldwide in 2010 [19].

**Figure 1:**
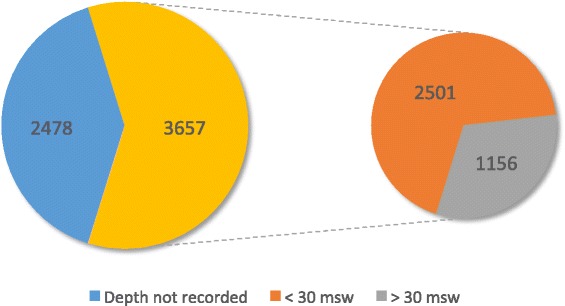
**Number of reported diving incidents (1999–2013).** Total number of diving incidents reported (left) in the period 1999–2013 in which the depth was not recorded (blue) or was recorded (yellow). Of those in which depth was recorded (right), the number of incidents involving dives to depths of less than 30 msw (orange) or greater than 30 msw (grey) is reported. Total number of reported incidents = 6,135. (Source: British Sub-Aqua Club incident database).

Data from the British Sub-Aqua Club annual incident report database do not, however, demonstrate the association of increased depth with a greater likelihood of accident or injury (Figure 1). However, from the same data set, it is not possible to ascertain the actual number of deep (>30 msw) and shallow (<30 msw) dives undertaken in the same time period. Data from other training agencies, however, indicate a bias in favour of shallow dives with around 70% of dives undertaken annually at depths of less than 30 msw [16]. Therefore, it is possible that that the incidents in dives with depths >30 msw actually represent a greater proportion of the incidents reported.

### Uptake of inert gas at increased environmental pressure

In order to appreciate the consequence of breathing gases under pressure, we must consider some gas laws. In the context of inert gas narcosis, we must consider Dalton’s and Henry’s law. Dalton’s law of partial pressures states that in a mixture of gases, the total pressure exerted is equal to the sum of the partial pressures of the individual gases [20]. Therefore, air (20.9% O_2_, 79.1% N_2_) at 1 ata total pressure is made up of oxygen at a partial pressure (*p*) of 0.209 ata and nitrogen at 0.791 ata. At depth, when the ambient pressures increase so do the partial pressures of the constituent gases (e.g. at 20 msw, the partial pressure of nitrogen in air is 3 × 0.791 = 2.373 ata). Originally devised in 1803 by William Henry, Henry’s law states that at a constant temperature, the amount of gas that dissolves in a given type and volume of liquid is directly proportional to the partial pressure of that gas in equilibrium with that liquid [20].

The consequence of these physical properties to the diver is that, when breathing gas under pressure, the constituents will dissolve in the body fluids (plasma, cytoplasm and lipids) proportional to the depth underwater since the alveolar/blood interface facilitates gaseous diffusion. Whilst the effects of high partial pressures of oxygen and other constituents of breathing gases should not be understated [11,21], a consequence of exposing tissues, particularly neurological tissue, to high partial pressures of nitrogen is narcosis [12].

### Signs and symptoms of inert gas narcosis

While, for most, the onset of symptoms of narcosis is associated with deeper dives (see Table 1), some individuals might be susceptible at shallower depths [22]. At depths of less than 30 msw, most symptoms are benign and, on the whole, hard to recognise (see Table 1) [12]. For instance, impairment of unrehearsed mental and physical tasks, such as sorting cards, is shown to be impaired as shallow as 10–20 msw [23]. Since symptoms tend to develop insidiously with depth, the onset of the more severe symptoms might render an individual incapable of self-control; and at >30 msw, the consequences could be catastrophic. Breathing compressed air at pressures exceeding 4 ata (30 msw), the equivalent of a *p*N_2_ ~ 3.5 ata, will invariably result in nitrogen narcosis [24,25]. At depths greater than 30 msw, symptoms can resemble those of alcohol, marijuana and some benzodiazepine drugs [26,27]. It is widely believed that the narcotic limit for diving on air is approximately 90 msw since studies to this depth have reported such severe symptoms of narcosis that individuals may find themselves completely incapacitated [28]. At these depths, however, when breathing air, the toxicity caused by the high partial pressure of oxygen would likely result in convulsions and drowning [21].

**Table 1:** **Signs and symptoms of nitrogen narcosis at different depths** [2,29]

**Depth (msw)**	**Atmospheric pressure (atm)**	***p*** **N2 (atm)**	**Signs and symptoms of narcosis**
0–10	1–2	0.79–1.58	Unnoticeable/minor symptoms such as subtle changes in behaviour
10–30	2–4	1.58–3.16	Mild impairment of unpractised tasks
			Impaired reasoning
30–50	4–6	3.16–4.74	Delayed response to visual and auditory stimuli
			Calculation errors and poor choices
			Mild amnesia
			Overconfidence, idea fixation and a sense of well-being
			Laughter (chambers) or anxiety (cold water)
50–70	6–8	4.74–6.32	Impaired judgement and confusion
			Hallucinations
			Delay in response to signals, instructions and other stimuli
			Uncontrolled laughter, hysteria (in chamber)
			Feelings of terror (in some)
70–90	8–10	6.32–7.90	Mental confusion
			Loss of memory
			Stupefaction and loss of judgement
90+	10+	7.90+	Hallucinations, increased intensity of vision and hearing
			Unconsciousness
			Death

Manual dexterity and reaction times appear to be affected with increasing depth, but it is unclear whether this is a direct result of neuromuscular deficit, cognitive dysfunction or the direct effect of pressure on the neurons [30,31]. Differential actions of inert gases and pressure on neuronal function might explain some of the discrepancies in *in vitro* and *in vivo* studies, supported by observations of high pressure neurological syndrome (HPNS) [24,32]. HPNS is a manifestation of neurological symptoms when exposed to very high pressures (>100 msw). Indications include headache and tremor, which are thought to be linked to enhanced release of the neurotransmitter serotonin since symptoms resemble those of serotonin syndrome and is likely to have a distinct action to narcosis [24,33,34]. Similarly, loss of balance control and the onset of vertigo have been observed at depth, often accompanied by tinnitus and hearing loss (neuro-vestibular). In the case studies reported, it is not clear whether IGN *per se* was responsible for the functional change or whether this was secondary to barotrauma [35]. At depths of between 30 and 50 msw, IGN affects central processing and it is believed that this is responsible for the amnesic effects of deep air diving [35-37]. Free recall, recognition of performed and verbal tasks as well as input into long-term memory are affected by even modest depths of 35 msw (the depth limit for most UK sport divers) [36,38-40]. Some studies also suggest that there are subtle, yet significant, changes to the arousal phase of the emotional response to stimuli when breathing pressurised air at narcotic depth [41]. In addition to cognitive function and coordination, it is suggested that other senses may be altered. The perception of pain is reduced by even modest depths [42] but, interestingly, thermal sensation does not appear to be changed by narcosis. The perception of comfort, however, is altered at depth such that a diver might feel less uncomfortable in colder conditions, thus risking hypothermia [43,44]. Visual impairment has been reported in some individuals [45-47]. Since depth is also usually associated with darkness and, in these circumstances, visual loss may be a compounding factor in incident manifestation when carrying out unpractised tasks [48].

In addition to increased depth, risk factors that can affect an individual’s susceptibility to IGN include fatigue and exertion [28], cold, increased partial pressure of CO_2_, hypercapnia [49], intoxication [50] and anxiety [22]. To mitigate the effects of IGN, ascent to a shallower depth is the simplest management. This will reduce the *p*N_2_ in the blood and tissues and reduce the narcotic symptoms. There is some evidence to indicate, however, that some of the symptoms of IGN can persist even when removed completely from the hyperbaric environment [51,52]. IGN can be prevented by avoiding diving to depths of >30 msw or by reducing the partial pressure of nitrogen in the breathing gas (by replacing some nitrogen with helium, which has no narcotic effect) [24,53].

**Figure 2:**
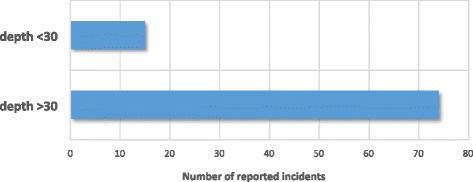
**Number of reported diving incidents involving narcosis (1999–2013).** Analysis of incidents known, or thought, to have involved narcosis, as reported by those involved at depths of less than or greater than 30 msw. Total number of reported incidents = 6,135. (Source: British Sub-Aqua Club incident database).

The precise role that the symptoms of narcosis play in diver injury or death is not clear, as the data required for such analysis are not always available (maximum depth is not consistently recorded following an incident) [7,16]. Overconfidence combined with confusion, neuromuscular incapacitation and cognitive decline are certainly contributing elements in diver injury or death at depth especially if current evidence as to the role of intoxication (by alcohol) in normobaric accidents is considered [27,50,54,55]. However, there are almost always other factors that will influence the outcome of a diving accident, more so at depth [4]. What is evident from the available data, however, is that depths of greater than 30 msw are associated with a 3.5-fold increase in the number of incidents known to involve narcosis (Figure 2), and that dives undertaken at depths >30 msw represent only 30% of all dives undertaken [16]. The incident records indicate that the common causal factors for diving-related injury are i) inadequate dive planning, ii) poor buddy checks, iii) failure to adequately monitor dive parameters during the dive, iv) diving beyond an individual’s personal capability, and v) lack of personal fitness which are discussed elsewhere [4,7,16]. However, in the context of this review, there are a disproportionate number of reported incidents associated with deeper dives.

### Mechanisms of action of IGN

Although the exact mechanism of IGN has not been fully elucidated, there are a number of experimentally supported theories as to its action, many of which are shared by those for other anaesthetics. There is no doubt that the site of action of narcosis in the brain is at the synapses, since inhaled anaesthetic agents, such as xenon (Xe) and nitrous oxide (N_2_O_2_), act at the level of the central nervous system and have both pre- and post-synaptic effects motor control [56-60]. The Meyer-Overton hypothesis suggests that the more lipid soluble an inhaled agent the more narcotic it is [61,62] (Table 2 shows the water and lipid solubility and relative narcotic potential of some gases). While this theory holds true for a number of inhaled anaesthetics, there are other factors that should be considered such as their interaction with synaptic surfaces, cellular proteins or the disturbance of metabolism in light of experimental evidence [63,64]. Physical hypotheses, such as disruption of lipid membranes, are attractive since they are simple to explain. Gaseous anaesthetics when solubilised in the lipid-rich membranes of neurons cause physical swelling on the membranes (up to 5%) leading to dysregulation of cell surface proteins and affect ion channel function which can be reversed, in part, by compression [56,65]. However, there are exceptions to this rule as not all narcotic agents change membrane thickness advocating a biochemical mechanism [66].

**Table 2:** **Relative narcotic strength of a number of gases** [12,22]

	**Solubility in solvent at 37°C (mg/ml)**		
**Gas**	**Water**	**Fat**	**Relative narcotic potency**
Helium (He)	0.009	0.015	0.2 (least narcotic)
Hydrogen (H_2_)	0.017	0.036	0.6
Nitrogen (N_2_)	0.013	0.067	1
Argon (Ar)	0.027	0.140	2.3
Xenon (Xe)	0.085	1.700	25.6 (most narcotic)
Oxygen (O_2_)	0.022	0.11	1.7
Carbon dioxide (CO_2_)	0.838	1.34	20.0

Anaesthetic agents such as hyperbaric nitrogen may bind competitively to cellular proteins, directly to ion channels or other hydrophobic sites within the cell [67,68]. Anaesthetic protein interactions occur that utilise hydrophobic pockets on protein surfaces through which the narcotic agent could interact. For example, xenon gas has been shown to occupy hydrophobic pockets within membrane proteins’ tertiary structure, which can inhibit their activity [69]. Protein kinase C (PKC), guanine nucleotide-binding proteins, GABA_A_ and ligand-gated ion channels on sensory and motor neurons have all been cited as target proteins for narcotic agents including nitrogen, although much of this work has been done in animal models or *in vitro* [56,70,71]. Whether these same biochemical mechanisms can be attributed to hyperbaric nitrogen has yet to be fully elucidated, but there is a growing body of evidence to support this notion. In experimental rats, the activity of inter-neuronal GABA_A_ receptors are desensitized during exposure to high partial pressures of nitrogen leading to decreased activity of the nigrostriatal pathway which is involved in coordination and is implicated in the symptoms of Parkinson’s disease [14,72,73]. Whilst it is likely that the actual mechanism of IGN action is probably multifactorial, the implication to those exposed to hyperbaric breathing gases is unchanged: The effects of IGN on the diver include disruption of neuromuscular function and will inevitably affect movement [8,12,30,74].

Most consider the effects of IGN to be an acute response to high partial pressures, which are alleviated by decreasing the partial pressure of the inert gas. However, there is evidence to indicate that some of the symptoms of IGN can be persistent. Following a single dive to 30 msw, measures of critical flicker fusion frequency (a measure of visual acuity) remained significantly altered 30 min after the dive [51]. This persistent alteration in function was reversed by treatment with 100% oxygen, indicating that some of the neurological alterations manifest by high partial pressures of nitrogen may be persistent similar to the delayed recovery from anaesthetics [75].

### Adaptation

The research is very limited with regard to adaptation and acclimatisation to nitrogen narcosis. Although it is evident that individuals’ functional deficit appears to be ameliorated during recurring exposures to the same pressures [76], it may be that the effects of narcosis experienced are worse upon arrival at depth. There may also be other mechanisms that compensate or exacerbate impairment of function. For example, metabolic challenges during diving may also alter a diver’s susceptibility to narcosis: during exertion underwater, there is likely to be increased plasma concentration of carbon dioxide (CO_2_) which has a narcotic index 20-fold higher than nitrogen (Table 2) [12]. Elevated alveolar CO_2_ associated with experimental hyperbaria has been proposed to be a sensitising factor in IGN [64].

There is little evidence from rigorous studies, however, to indicate that any specific physiological adaptation to nitrogen narcosis occurs [76]. Studies in laboratory animals have, thus far, failed to demonstrate any improvement or change in neurochemical or behavioural indices of narcosis following repeated exposure to high nitrogen partial pressures [72]. Human studies are, unfortunately, less prevalent but there is no evidence to indicate any physiological adaptation especially when considering reaction times and coordination and other objective measures made in subjects exposed to repetitive dives to depth [77]. Learned tasks, however, have been shown to become easier on subsequent exposure, which suggests a degree of ‘habituation’, rather than true ‘adaptation’, which is also observed in normobaric conditions [77,78].

Some studies have concluded that the rate of increase of pressure can affect the onset or severity of IGN experienced, with slower onset of pressure of inert gas corresponding to less narcosis [67]. This could be due to a degree of short-term adaptation or compensation. Little is known about the ability of the body to acclimatise to deep dives. There is plenty of anecdotal evidence from divers (the author included) that narcosis is felt less towards the end of a series of repetitive dives to depth or at the end of the diving season (the term “dived up” is often used in the diving community).

## Conclusions

SCUBA diving is an increasingly popular pastime for many, but descending to depth can present a number of risks even to experienced divers; and with increasing depth is the likelihood that the diver will suffer from IGN. While breathing air at depths of >30 msw, IGN will affect all divers and the effects can be incapacitating.

Evidence indicates that working-up to deeper diver progressively and prior hyperbaric exposure may help to reduce this risk on an individual. Frequent practice of tasks required at depth (such as rescue skills and use of life-saving equipment) might be of benefit to a diver exposed to narcosis, and a pragmatic approach to deep diving should include personal assessment of “fitness to dive” as well as avoiding the predisposing factors described earlier [9]. Without doubt, however, suitable training, practice and diving buddy selection can help alleviate some of the risk.

## Abbreviations

IGN: inert gas narcosis
msw: metres of sea water (depth)
atm: atmospheres of pressure
bar: barometric pressure (1 bar = 1 atm = 750.06 mmHg)
N_2_: nitrogen gas
O_2_: oxygen gas
CO_2_: carbon dioxide gas
N_2_O: nitrous oxide gas
SCUBA: self-contained underwater breathing apparatus
GABA_A_: gamma-aminobutyric acid receptor
